# Enhanced capabilities for multi-crystal data collection based on double mesh scans

**DOI:** 10.1107/S2059798325005145

**Published:** 2025-06-26

**Authors:** Igor Melnikov, Olof Svensson, Gleb Bourenkov, Alexander Popov

**Affiliations:** a European Synchrotron Radiation Facility BP 220 38043Grenoble France; b European Molecular Biology Laboratory Hamburg Outstation, Notkestrasse 85 22607Hamburg Germany; Stanford University, USA

**Keywords:** *Dozor*, *Dozor-m*2, *Resheteau*, macromolecular crystallography, X-ray mesh raster scans, sample localization, data-collection strategies

## Abstract

Two-dimensional X-ray mesh (raster) scans are broadly used in macromolecular crystallography to identify positions on the sample holder at which diffraction images can be collected. Based on a single mesh scan, the commonly known method *Mesh&Collect* allows the collection of small angular wedges of diffraction data from multiple crystals identified in the mesh-scan area. Recently, the capabilities of the method have been extended by using two mesh scans instead of one. The scans are usually performed at orthogonal orientations of the rotation axis, or any other angular difference less than 90° can be used. This allows the more accurate determination, when compared with a single mesh scan, of the 3D centre positions of each crystal contained in the scanned area and gives better estimates of their dimensions and diffraction qualities. We present the upgraded *DoubleMesh&Collect* method based on the *Dozor*, *Dozor-m*2 and *Resheteau* software programs which automatically analyse the diffraction images of the two mesh scans. The applicability of the method is demonstrated on several cases, including room-temperature diffraction studies. The problem of cartography of large crystals is discussed.

## Abstract 2

A method for multi-crystal data collection and the software programs *Dozor*, *Dozor-m*2 and *Resheteau* for the detection of individual crystals, the determination of their positions on a sample holder and the estimation of their dimensions and shapes based on double raster X-ray scans are presented.

## 1. Introduction

A standard diffraction data-collection experiment in macromolecular crystallography (MX) implies rotation of the sample to measure the integral diffraction intensities. The point around which sample rotation will be carried out can be selected using an on-axis optical microscope or X-ray mesh (raster) scans. During an X-ray mesh scan, the sample holder is translated under exposure of the X-ray beam and diffraction images are recorded at each point of a pre-defined two-dimensional grid. The images are then analysed [*i.e.* using *DISTL* and *Spotfinder* (Zhang *et al.*, 2006 ▸), *CrystFEL* (White *et al.*, 2012 ▸), *Dozor* (Svensson *et al.*, 2015 ▸; Zander *et al.*, 2015 ▸) *etc.*] for the presence of diffraction. The diffraction is subsequently ranked, giving a score for each image, and the results are displayed in a two-dimensional (2D) heat map.

Compared with optical localization, X-ray mesh scans can directly point out well diffracting regions of a sample holder (Hirata *et al.*, 2019 ▸; Song *et al.*, 2007 ▸; Svensson *et al.*, 2015 ▸). Nevertheless, the choice of optimal rotation centre in 3D using a diffraction heat map is not straightforward. In the simplest case there is only one isotropic crystal inside the crystal holder; its diffracting regions are represented by a nonzero score on the corresponding heat map for a given mesh scan. One can choose a point on such a 2D map (usually simply represented by the median, or centre of mass, of the score distribution) to be centred in the beam. To find the third centring coordinate (*i.e.* depth, in the direction perpendicular to the mesh-scan plane), the crystal is rotated by 90° and a linear diffraction scan (perpendicular to the rotation axis) is performed. The third centring coordinate is then determined as the median of the linear scan diffraction-score distribution. This procedure is commonly known as ‘X-ray centring’.

In our previous publications, we have presented methods and the software programs *Dozor* and *MeshBest* (Melnikov *et al.*, 2018 ▸) for 2D mesh-scan analysis. *Dozor* detects the presence of protein crystal diffraction in each particular image, giving it a score, and then builds a 2D heat map of the score values. It also gives a list of diffraction-spot centre coordinates in each image. Based on this map and the given spot coordinates from *Dozor* analysis, *MeshBest* distinguishes regions belonging to the same (individual) crystals and detects regions where several crystals have been exposed to the beam simultaneously, producing multi-crystal diffraction patterns. Finally, it converts the 2D diffraction-score heat map to a 2D crystal map showing estimates of the dimensions, 2D centre positions and diffraction qualities of each crystal contained in the mesh-scan area. To be consistent with *Dozor*, *MeshBest* has been rewritten in Fortran and has become *Dozor-m*2. The programs are implemented in the X-ray centring workflow, which is accessible through the beamline experiment management software *MXCuBE* (Oscarsson *et al.*, 2019 ▸), and have successfully been used during the last several years, especially for completely automatic data collection (Bowler *et al.*, 2015 ▸).

In practice, crystal-harvesting techniques may not always yield samples containing a single crystal; when multiple crystals are contained in the sample holder their 3D X-ray centring as described above is obstructed by the ambiguity in centring choices. A mesh scan may choose one crystal, and the subsequent linear scan 90° apart may choose a different crystal for the third centring coordinate, because there is no cross-checking of crystal identity between the scans. To avoid this issue, but still be able to collect data, it might be possible to use a flat sample holder in which the third coordinate is determined by edge centring. Such a method, however, lacks a certain amount of accuracy and any information about diffraction-quality distribution across the third coordinate. Another alternative had been proposed, a method named *Mesh&Collect* (Zander *et al.*, 2015 ▸), to centre each crystal in the beam using 2D mesh-scan coordinates (without precise centring in the third direction, parallel to the beam) and collect small wedges (5–10°) so that each crystal would still stay in the beam, relying on the fact that the sample thickness is not large. The small wedges of data could then be merged together, optionally applying a selection algorithm. This method has the advantage that it allows a reduction in radiation damage per complete data set as the dose is distributed over many crystals.

Recently, we have managed to resolve the ambiguity in centring choice for multiple crystals by using two 2D mesh scans (a double mesh scan) performed at different orientations and subsequent automatic analysis by software. The programs *Dozor* and *Dozor-m*2 execute the already established procedure of finding the 2D positions of individual crystals in each of the two mesh scans. However, the core of the newer method is a conjunction between *Dozor*, *Dozor-m*2 and the new program *Resheteau*, which serves to identify the correspondence between crystal areas in different scans and can, finally, provide optimal 3D centring and optimal beam-size selection (if selectable) for multi-crystal data collection. As a result, the new *DoubleMesh&Collect* method allows the measurement of wider wedges (up to 360°) for each crystal. The sample rotation between the two mesh scans can be substantially less than 90°, which is especially important in MX experiments with restrictions on rotation range, for example in measurements using crystallization plates or chips. The *DoubleMesh&Collect* approach should provide information about 3D positions for all crystals inside the sample holder and thus allow the rational organization of completely automatic multi-crystal data collection. However, there are fundamental limitations to the method which relate to the inherent diffraction properties of large crystals. A description of the method, proof of concept using several test cases and a discussion of the applications of the method can be found below.

## 2. Methods

The described method is incorporated as *DoubleMesh&Collect* into the workflow-execution system used at the ESRF MX beamlines (De Nolf *et al.*, 2024 ▸). The workflow manages the execution of the mesh scans and the subsequent sequential analysis by *Dozor*, *Dozor-m*2 and *Resheteau*. It is invoked through the *MXCuBE* interface (Oscarsson *et al.*, 2019 ▸) by manually or automatically defining a grid for X-ray scanning across the sample area at two goniometer orientations. The processing results can be tracked via the ESRF Data Portal on the web.

### 2.1. Initial mesh-scan analysis by *Dozor*

**Figure 1:**
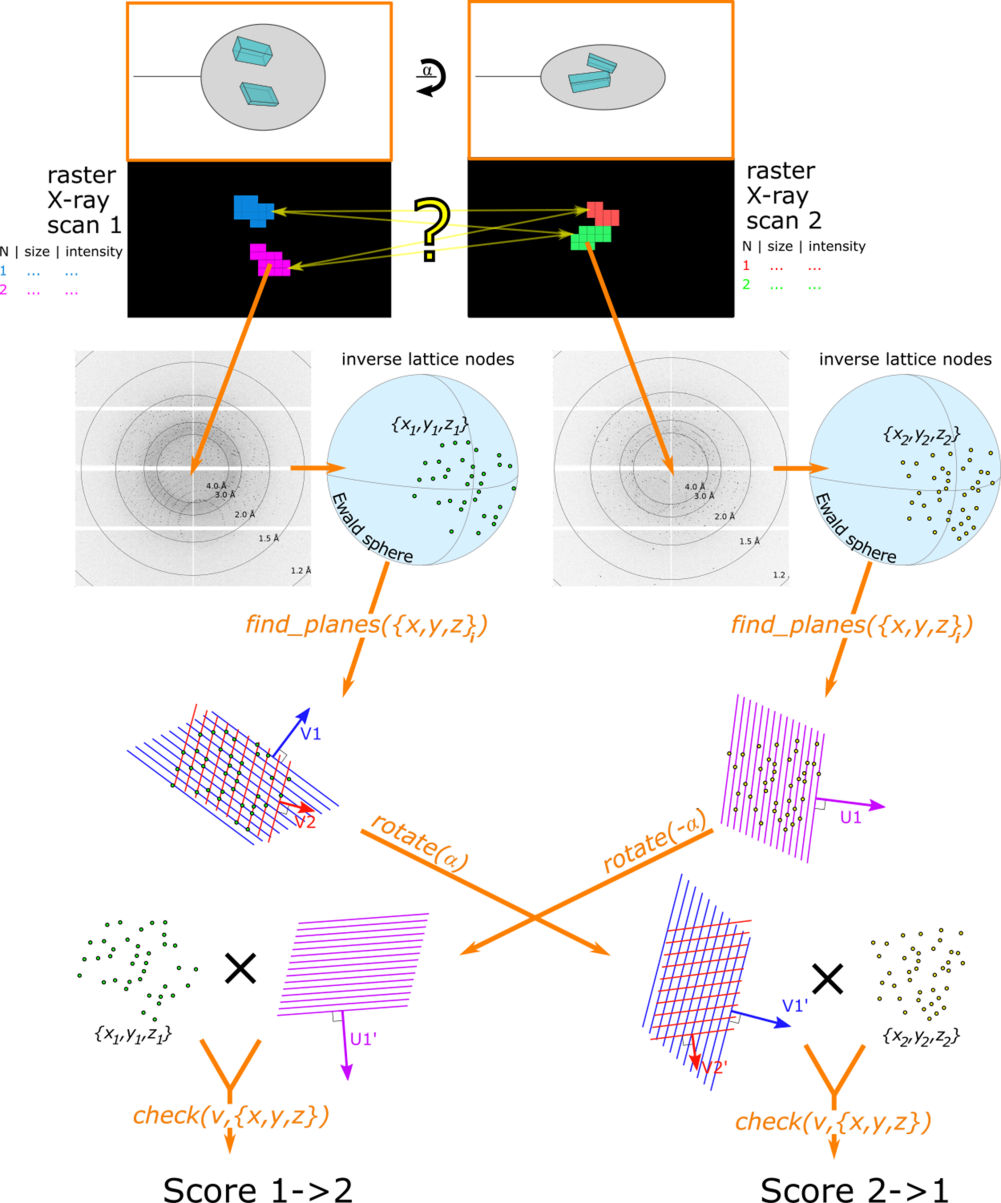
Overview of the workflow of the method. (*a*) Two mesh scans are performed over the sample area at different orientations of the rotation axis (with angular difference α). Analysis by *Dozor* and *Dozor-m*2 showed two crystals (pictured in different colours) in each of the mesh-scan areas. The yellow question mark indicates ambiguity in the selection of crystal-centring coordinates. (*b*) Illustration of the algorithms used by *Resheteau*. Raw images are analysed by *Dozor*; the spot coordinates are then used by *Resheteau* to reconstruct lattice subsets in reciprocal space. The function find_planes() takes as its argument the 3D coordinates of lattice points and returns plane-vector coordinates (*e.g.V*1, *V*2 in the first case, *U*1 in the second case) based on these points. The vectors are then rotated accordingly by the angular difference α and applied to the opposing crystal point coordinates using the function check(), which returns a score (see Appendix *A*[App appa]). Finally, the scores from different plane vectors are compared to obtain a maximum score as a final match score between crystals.

**Figure 2:**
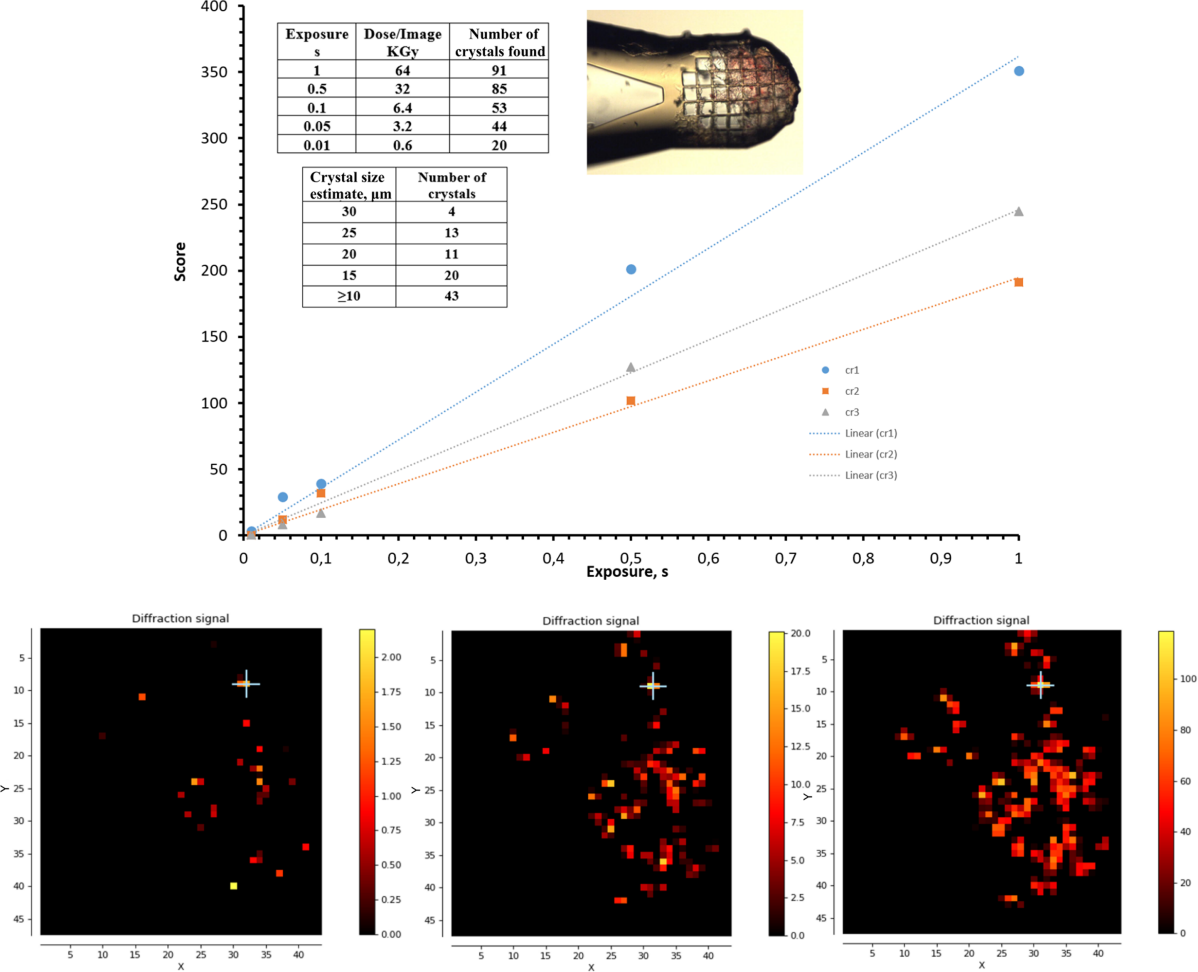
Test measurements to demonstrate the sensitivity of the method and diffraction signal score linearity. Top: the score estimates for three most strongly diffracting crystals (cr1, cr2 and cr3, all approximately 30 µm in size) show good linear proportionality versus exposure time; the two tables describe the experimental conditions, and a snapshot of the crystal holder as mounted on the goniometer is also shown. Bottom: three *Dozor*-score heat maps of the mesh scans with exposure 0.01, 0.1 and 1.0 s, respectively. *X* and *Y* are the two axes of the scan; *X* is parallel to the goniometer rotation axis, whereas *Y* is the laboratory vertical axis. Units on the axes are scan steps, which are equal to the beam size (10 µm).

The schematic workflow is presented in Fig. 1 ▸ and comprises several successive steps. The crystal holder is mesh-scanned two times at different rotation angles while keeping the size and position of the scan grid the same. As the measurement progresses, each of the images is analysed by *Dozor*, which enables the automatic detection of diffraction from macromolecular crystals and estimates its quality. The program delivers rapid diffraction-pattern recognition and has high sensitivity (see the examples in Fig. 2 ▸). Since 2014, *Dozor* has successfully been used on several synchrotron MX beamlines as a part of the control data-collection software (Svensson *et al.*, 2015 ▸; Melnikov *et al.*, 2018 ▸; Gao *et al.*, 2018 ▸; Oscarsson *et al.*, 2019 ▸). The main principles that the program is based on have been described in Zander *et al.* (2015 ▸). *Dozor* produces estimates of diffraction signal, mean background intensity, a list of diffraction-spot coordinates and their partial intensities in each image. This information is written into corresponding files.

### 2.2. *Dozor-m*2

Each mesh scan is individually analysed by *Dozor-m*2, the program which inherits the algorithms from *MeshBest* that have been described in Melnikov *et al.* (2018 ▸). Mesh-scan images ranked by *Dozor* are checked for the presence of multiple diffraction patterns superimposed in the same image by analysing diffraction difference vector statistics. The diffraction images containing only a single-crystal diffraction pattern are then selected and grouped into those belonging to the same crystal by proximity of spot positions in those images. Hierarchical cluster analysis based on such similarity between images is used to dissect the mesh-scan area into interconnected regions that can be treated as being from individual crystals. In the next step, *Dozor-m2* creates a map in which each grid point of the scan is labelled with a number denoting a particular crystal or with zero if diffraction has not been found. Numbers for individual crystals are assigned in ranking accordance with their diffraction-signal strength. The program also produces a list of sizes and centre positions for all crystals found in the area and estimates the optimal beam size to be used for each.

After processing both mesh scans, *Dozor-m*2 finds all geometrically possible (by invariance of coordinates along the rotation axis) pair combinations of crystal regions between the two scans that could represent the same crystal. It provides the 3D centring coordinates for all potential pairs. For the 3D coordinate grid tied to the first scan, in which the *X* axis coincides with the beam direction in the first scan and the *Z* axis coincides with the axis of rotation, the coordinates of the crystal are calculated as
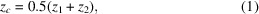

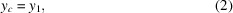

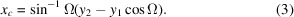
Here, *y*_*i*_ and *z*_*i*_ are the coordinates in the 2D grid of the *i*th scan (*i* = 1, 2) separated by the angle of rotation Ω. In the last step, the new program *Resheteau* checks whether all of these potential pairs are truly from the same crystal or not and gives the final list with centring positions (Section 2.3).

### 2.3. *Resheteau*: verifying that diffraction images taken at different sample orientations are truly from the same crystal

*Resheteau* is new software which examines whether the diffraction patterns produced in the two mesh scans are consistent in their difference in the rotation-axis orientation, a condition which guarantees that the crystal producing these patterns is the same one. The diffraction patterns are analysed by first reconstructing a reciprocal-lattice subset from spot positions. The next step involves determining whether these reciprocal-lattice constructs are related via the known angular difference of the rotation-axis positions at which the mesh scans were performed. The following paragraphs provide further details about each step of the method.

#### 2.3.1. Reciprocal-lattice subset construction

Images attributed to each crystal in a mesh-scan area are selected for further analysis (see Fig. 1 ▸). Diffraction spots obtained from these images are gathered: their 2D positions, partial intensities and background estimates are listed. The spots are used to reconstruct the reciprocal-lattice nodes by taking the Ewald sphere geometric representation. The next step taken by *Resheteau* is to search for periodicity in this set of 3D points (a subset of the reciprocal lattice located on the Ewald sphere surface).

#### 2.3.2. Searching for periodicity in a reciprocal-lattice subset

Similarly to the Rossmann indexing algorithm (Steller *et al.*, 1997 ▸), in the given set of 3D node positions derived from the image, *Resheteau* tries to find parallel periodic planes in which the nodes should be arranged (see Fig. 1 ▸*b*). For this, an isotropic set of unit direction vectors is created in spherical coordinates. For each direction vector, projection coordinates of all the nodes onto the vector axis are calculated. A histogram of projection coordinates is next calculated. A fast Fourier transform (FFT) is performed on the obtained histogram in order to see periodicity in coordinate arrangement. To normalize the Fourier spectra, a correction is applied to the histogram prior to FFT calculations by subtracting a slowly moving average from the histogram and dividing it by the square root of the moving average (see Appendix *A*[App appa]). This results in Fourier magnitudes that should asymptotically follow a χ-distribution (with two degrees of freedom, also known as a Rayleigh distribution; see Appendix *A*[App appa]) if there is no periodicity in the histogram. Finally, having an outlier peak in a Fourier spectrum would indicate a periodicity in the coordinates of the node projections. *Resheteau* selects several direction vectors with the highest Fourier peak values and performs fine refinement of their directions. If the Fourier peak value increases above 7 [(1–2 × 10^−11^) quantile of the χ-distribution] during such refinement, the corresponding direction unit vector is transformed to a plane vector by multiplying it by the spatial frequency of the Fourier peak. As a result, a set of unique plane vectors is produced for each crystal in each mesh scan.

### 2.4. Validating crystal correspondence in two scans

Diffraction patterns recorded from the same single crystal but at different angles must be subsets of the same reciprocal lattice but sliced at the corresponding different angular orientations. This means that the periodic arrangement of nodes in reciprocal space must be conserved no matter which subset of the reciprocal lattice is taken using the Ewald sphere construct. Consequently, the plane vectors (Section 2.3.2) calculated by *Resheteau* for a crystal in the first mesh scan must also serve as its plane vectors in the second mesh scan (if rotated by the angular difference between the scans), and vice versa.

*Resheteau* rotates the plane vectors calculated for crystals in the first mesh scan forwards by the angular difference and the plane vectors calculated for crystals in the second mesh scan backwards by the angular difference (see Fig. 1 ▸*b*). Then, for all potential crystal pairs between two scans the rotated plane vectors are questioned as also being plane vectors for the other crystal in the pair (see Fig. 1 ▸*b*). *Resheteau* applies the function check(), which examines whether the rotated plane vector fits the diffraction of the other crystal. This function calculates projection coordinates of the reciprocal-lattice nodes (previously already obtained for the other crystal; see Section 2.3.1) onto the rotated plane vector axis. This is followed by histogram construction and FFT analysis, as described in Section 2.3.2. The peak in the Fourier spectrum is expected to be at the concrete spatial frequency given by the rotated vector length. Peak values above 5 [(1–4 × 10^−6^) quantile of the χ-distribution; see Section 2.3.2, Appendix *A*[App appa]) are used as a quantitative indicator for matching of the plane vector and the diffraction pattern of the other crystal. The binary decision of matching between the two crystal areas is made based on the maximum Fourier peak value across all (plane vector)–(diffraction pattern) pairs used in the function check().

## 3. Test results

All test cases were measured on the ID23-1 beamline (Nurizzo *et al.*, 2006 ▸) at the ESRF equipped with a Dectris EIGER2 X 16M detector. X-ray apertures from 10 to 50 µm were used for data collection in accordance with the calculated crystal sizes. Protein crystals of MAR (Ghai *et al.*, 2013 ▸; Bukhdruker, 2023 ▸), BR (Bukhdruker, 2023 ▸) and myoglobin from equine skeletal muscle (Sigma–Aldrich) were kindly provided by collaborators. All absorbed X-ray radiation dose values were calculated using *RADDOSE*-3*D* (Zeldin *et al.*, 2013 ▸).

### 3.1. *Dozor* sensitivity

A test case to estimate the sensitivity of *Dozor* is presented in Fig. 2 ▸. About a hundred crystals of the membrane protein MAR (a light-driven proton pump from *Candidatus* Actinomarina minuta expressed in *Escherichia coli*) of different sizes from 5 to 30 µm were harvested on a micromesh sample holder (MiTeGen), cryocooled to 100 K and mounted on the goniometer. An X-ray beam of 10 µm in FWHM in both directions was used, attenuated to 10%, resulting in a flux of 1.7 × 10^10^ photons s^−1^; the synchrotron current was 38 mA and an X-ray energy of 14.4 keV was used. The sample area was mesh-scanned five times with a different exposure time per image from 0.01 to 1.0 s (keeping the same beam transmission). The best diffraction resolution from these crystals was around 1.8 Å. Each of the images was processed by *Dozor*, with further mesh-scan analysis carried out by *Dozor-m*2. The maximum number of recognized individual crystals is 91 for 1.0 s of exposure time (dose per image 64 kGy); more than half of this number were found using 0.1 s of exposure time and 20 crystals were detected with an exposure time of 0.01 s (dose per image 0.6 kGy; see Fig. 2 ▸). The *Dozor* scores calculated for the top three crystals with strongest diffraction show a good linear fit versus exposure time.

### 3.2. *DoubleMesh&Collect* approach

The double mesh-scan method extends the existing approach (*Mesh&Collect*; Zander *et al.*, 2015 ▸) of collecting data from multiple crystals mounted in the same sample holder. Compared with *Mesh&Collect*, it allows the optimal centring of crystals in 3D, thus lifting the restriction of the method on rotation angle which came from 2D-centring. In addition, the new method ensures rational sorting of crystals taking into account their homogeneity and diffraction strength thanks to *Dozor-m*2 analysis. The latter is especially useful when organizing measurements from large crystals that consist of disoriented blocks (Section 3.2.3). It can also help to extract the best possible data from crystals of poor quality. In the next sections, we show some test cases for demonstration purposes.

#### 3.2.1. *DoubleMesh&Collect* under cryogenic conditions

**Figure 3:**
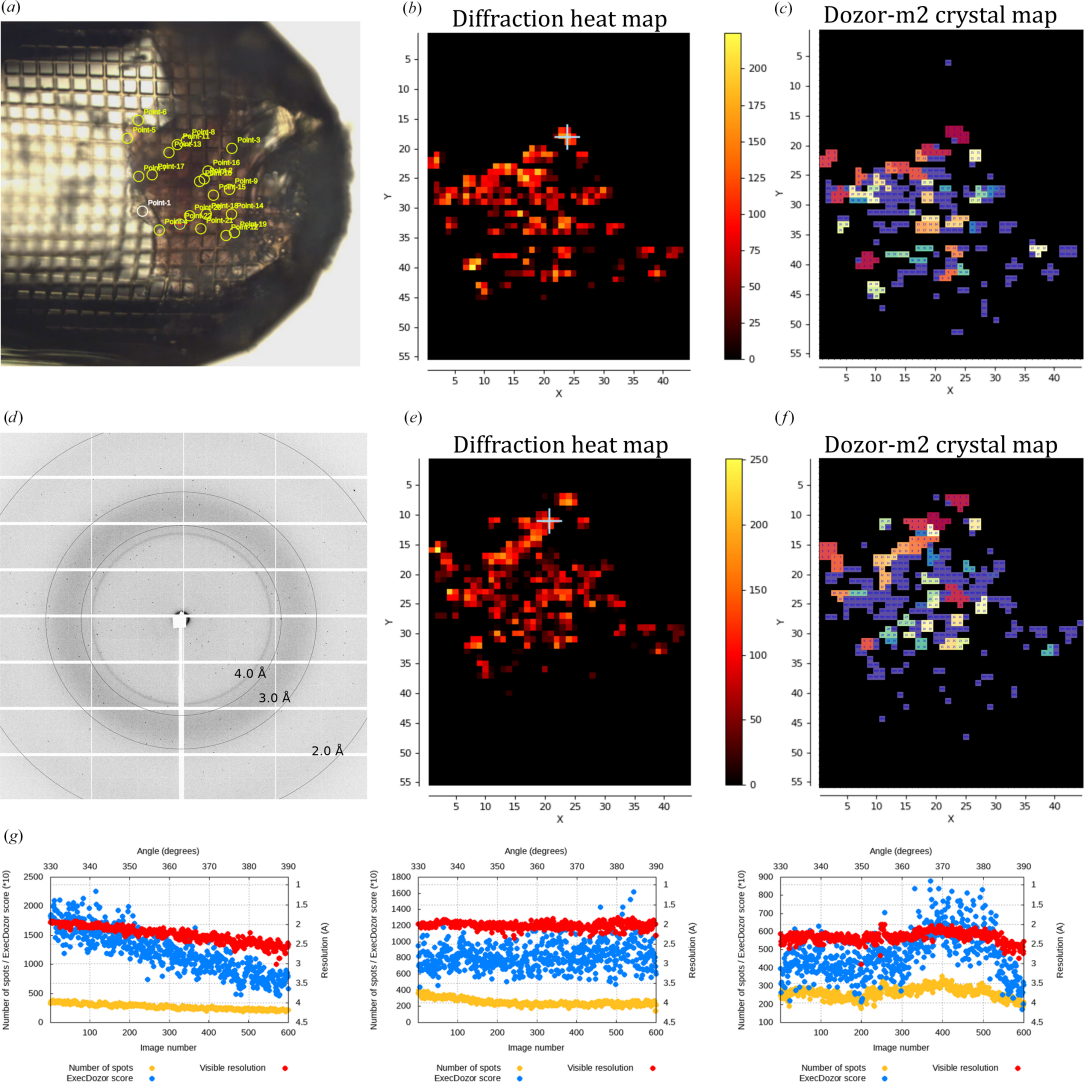
Multi-crystal data collection from crystals of the membrane protein MAR. (*a*) A snapshot of a mesh micromount sample holder with the position marks for the crystals that were selected for data collection. (*b*, *e*) Two diffraction heat maps of the mesh scans separated by 60° of sample rotation. *X* and *Y* are the two axes of the scan; *X* is parallel to the goniometer rotation axis, whereas *Y* is the laboratory vertical axis. Units on the axes are scan steps, which are equal to the beam size (10 µm). (*c*, *f*) Two crystal maps for the mesh scans generated by *Dozor-m*2. Different colours are used to show distinct crystal areas, which are arbitrarily numbered, and the numbers are shown for each pixel; regions with overlapping diffraction patterns or contaminated by salt diffraction are shown in violet and numbered 999. (*d*) A sample diffraction image from the mesh scans. (*g*) Data-collection plots for three selected crystals.

Crystals of the membrane protein MAR (a light-driven proton pump from *Candidatus* Actinomarina minuta) with sizes ranging between 10 and 30 µm were harvested in a micromesh sample holder (Fig. 3 ▸) and two mesh scans were performed separated by a 60° turn of the rotation axis. A beam aperture of 10 µm was used for the scans and the exposure time was 0.01 s per image (the dose per image was 40 kGy). *Dozor-m*2 found 43 and 37 individual crystals in the first and second scan, respectively. It also determined 93 possible pair combinations between the crystals of the two scans, and from these *Resheteau* selected 18 positions to be truly matching crystals. Subsequently, 60° of rotation data were collected from all of these positions in a ranking order provided by the integral diffraction score of *Dozor-m*2. Each data set was collected using a beam size chosen by the program in accordance with the crystal sizes. The diffraction images from each partial data set were processed with *Dozor*, *XDS* and *XSCALE* (Kabsch, 2010 ▸). Two data sets were excluded by the clustering algorithm (Giordano *et al.*, 2012 ▸). The final data set obtained by merging 16 partial data sets is presented in Table 1 ▸ and extends to a resolution of 1.9 Å.

#### 3.2.2. *DoubleMesh&Collect* under room-temperature conditions

**Figure 4:**
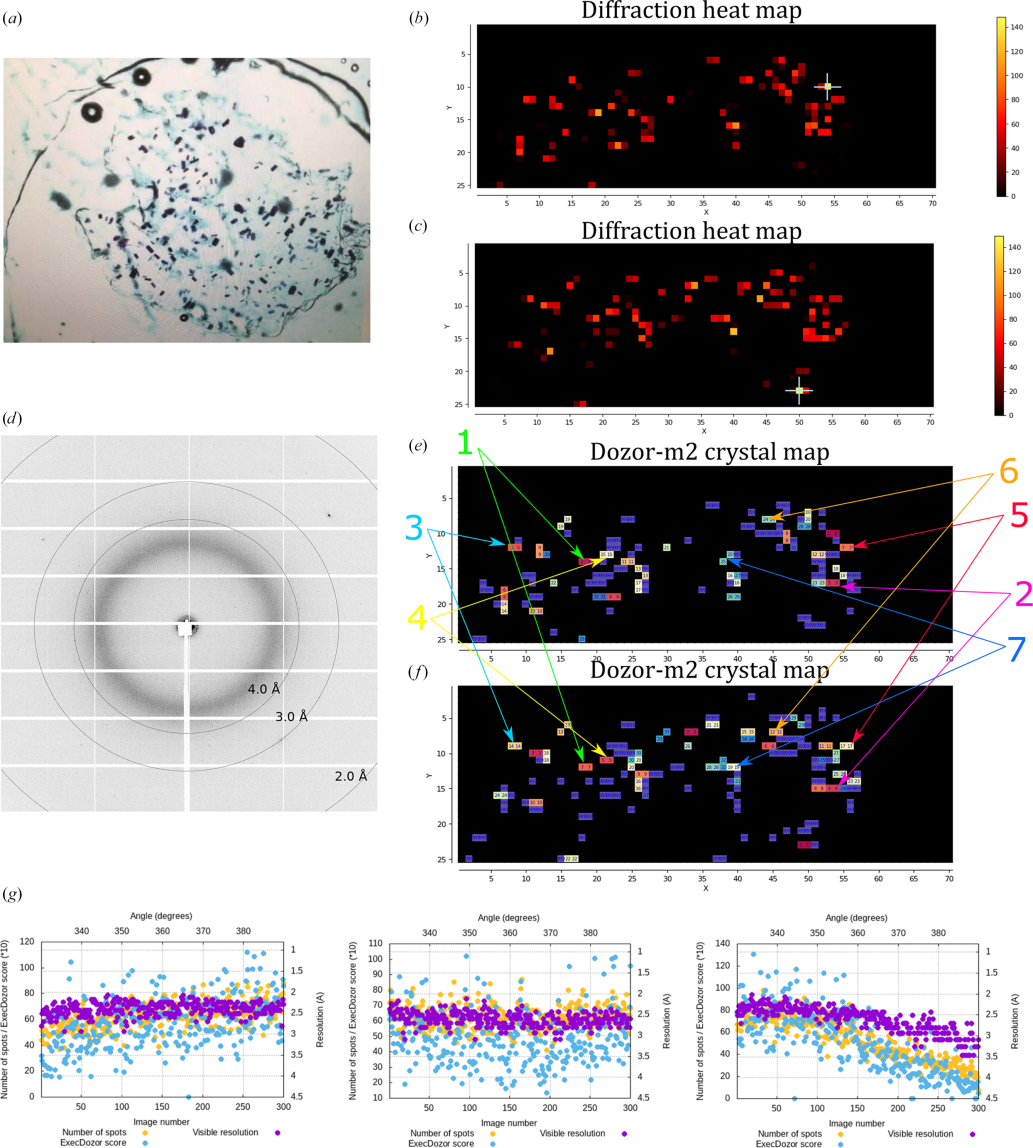
Multi-crystal data collection from crystals of the membrane protein BR. (*a*) A snapshot illustrating crystalline sample sandwiched between two Mylar foils. (*b*, *c*) Two diffraction heat maps of the mesh scans separated by 60° of sample rotation. *X* and *Y* are the two axes of the scan; *X* is parallel to the goniometer rotation axis, whereas *Y* is the laboratory vertical axis. Units on the axes are scan steps, which are equal to the beam size (30 µm). (*c*, *f*) Two crystal maps for the mesh scans generated by *Dozor-m*2. Different colours are used to show distinct crystal areas, which are arbitrarily numbered, and the numbers are shown for each pixel; regions with overlapping diffraction patterns or contaminated by salt diffraction are shown in violet and numbered 999. (*d*) A sample diffraction image from the mesh scans. (*e*, *f*) Two crystal maps for the mesh scans produced by *Dozor-m*2. Crystals from which data were collected are indicated by arrows. (*g*) Data-collection plots for three selected crystals.

Crystals of the membrane protein bacteriorodopsin (BR) with sizes ranging between 20 and 40 µm were mounted in a custom holder carrying a two Mylar foil sandwich (Fig. 4 ▸) and two mesh scans were performed separated by 60° of sample rotation. A beam aperture of 30 µm was used for the scans, the flux was 4.5 × 10^12^ photons s^−1^, the exposure time per image was 0.01 s and the beam transmission was 40% (with a dose per image of ∼10 kGy). *Dozor-m*2 found 32 and 36 individual crystals in the first and the second scan, respectively, and determined 48 possible pair combinations between the crystals in the two scans; from these, *Resheteau* selected seven positions to be truly matching crystals. Subsequently, 60° of rotational data using 2% of beam transmission and an exposure time of 0.01 s per image were collected from all of these positions. The total dose absorbed by each crystal was ∼0.3 MGy (estimated using *RADDOSE*-3*D*; Zeldin *et al.*, 2013 ▸). The diffraction images from each partial data set were processed, scaled and merged with *XDS* and *XSCALE* (Kabsch, 2010 ▸). All seven data sets were processed successfully and their statistics are presented in Table 2 ▸. The final combined data set has 100% completeness and extends to a resolution of 1.8 Å.

#### 3.2.3. Data collection from a crystal conglomerate

**Figure 5:**
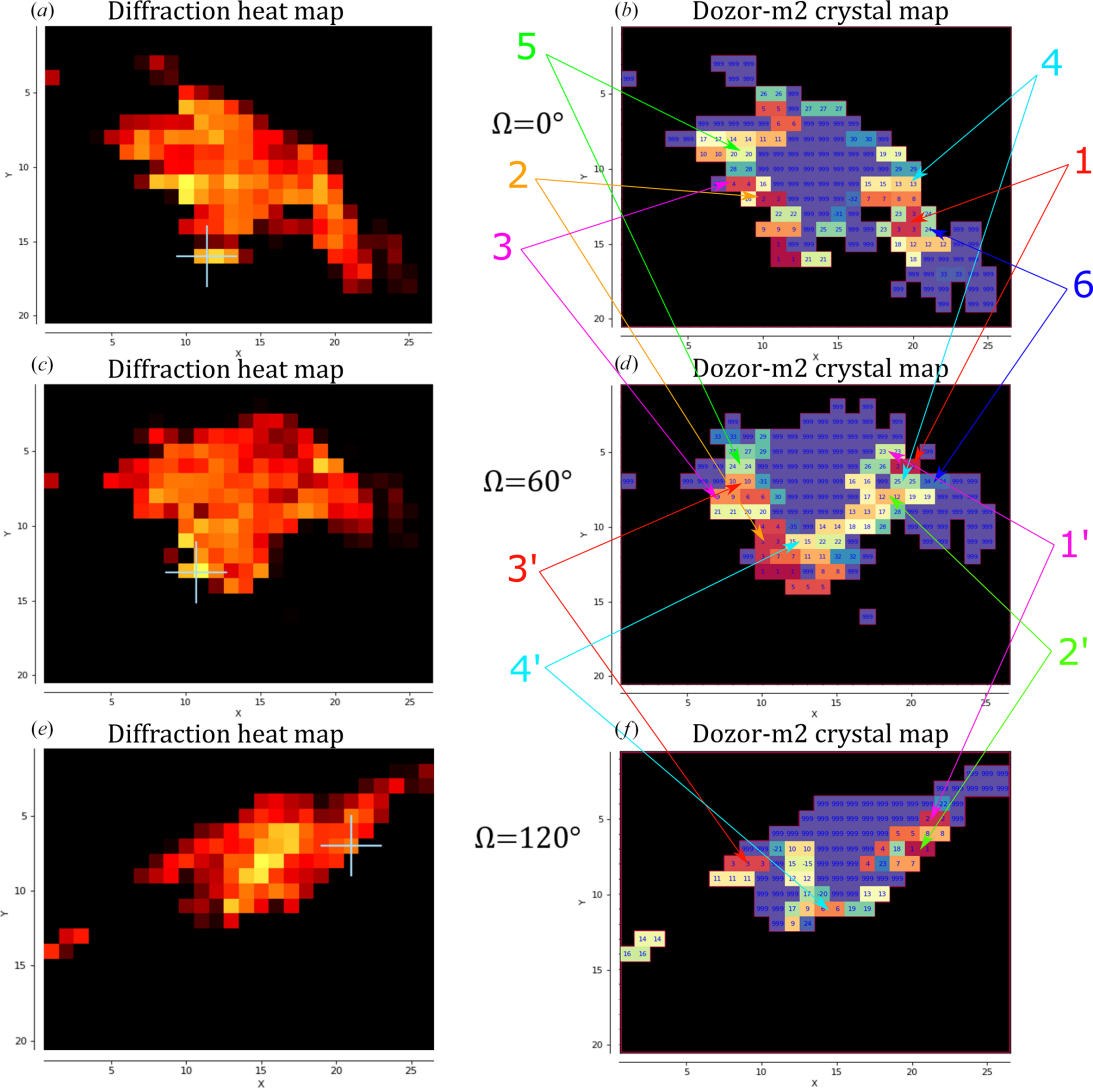
Multi-crystal data collection from a horse myoglobin crystal conglomerate using two double mesh scans in the ranges 0–60° and 60–120°. The diffraction heat maps of the mesh scans are shown on the left (*a*, *c*, *e*), while the crystal maps produced by *Dozor-m*2 are shown on the right (*b*, *d*, *f*). *X* and *Y* are the two axes of the scan; *X* is parallel to the goniometer rotation axis, whereas *Y* is the laboratory vertical axis. Units on the axes are scan steps, which are equal to the beam size (10 µm). (*c*, *f*) Two crystal maps for the mesh scans generated by *Dozor-m*2. Different colours are used to show distinct crystal areas, which are arbitrarily numbered, and the numbers are shown for each pixel; regions with overlapping diffraction patterns or contaminated by salt diffraction are shown in violet and numbered 999. Crystals from which data were collected are indicated by arrows.

The application of double mesh scanning can be crucial for diffraction measurements in cases where the test sample consists of highly disoriented crystal blocks or is a complex mixture of protein crystals and salts. In such cases, it is difficult to obtain a complete data set when selecting only one sample position. Here, it is possible to use the *DoubleMesh&Collect* method several times at different angular positions of the sample. As an example of this approach, we present the data-collection procedure from a crystal sample of horse myoglobin which is a crystalline conglomerate consisting of a mixture of highly disoriented plates. The *DoubleMesh&Collect* method was used twice in angular position combinations for scans of 0–60° and 60–120° (Fig. 5 ▸). As a result of the first double mesh scan, six positions were selected by *Resheteau* from 155 possible positions determined by *Dozor-m*2. Based on these positions, six rotational data sets in the range 0–60° were collected to a resolution of 1.6 Å. In the second double mesh scan four positions were selected from 85 possible combinations and four rotational data sets in the range 60–120° were subsequently collected. All ten of these data sets were processed successfully by* XDS*, four of which (two from the first run and two from the second run with better statistics) were scaled and merged by *XSCALE.* The completeness of the merged data set is 96.5% (Table 3 ▸).

#### 3.2.4. Centring a crystal diffracting to moderate resolution

**Figure 6:**
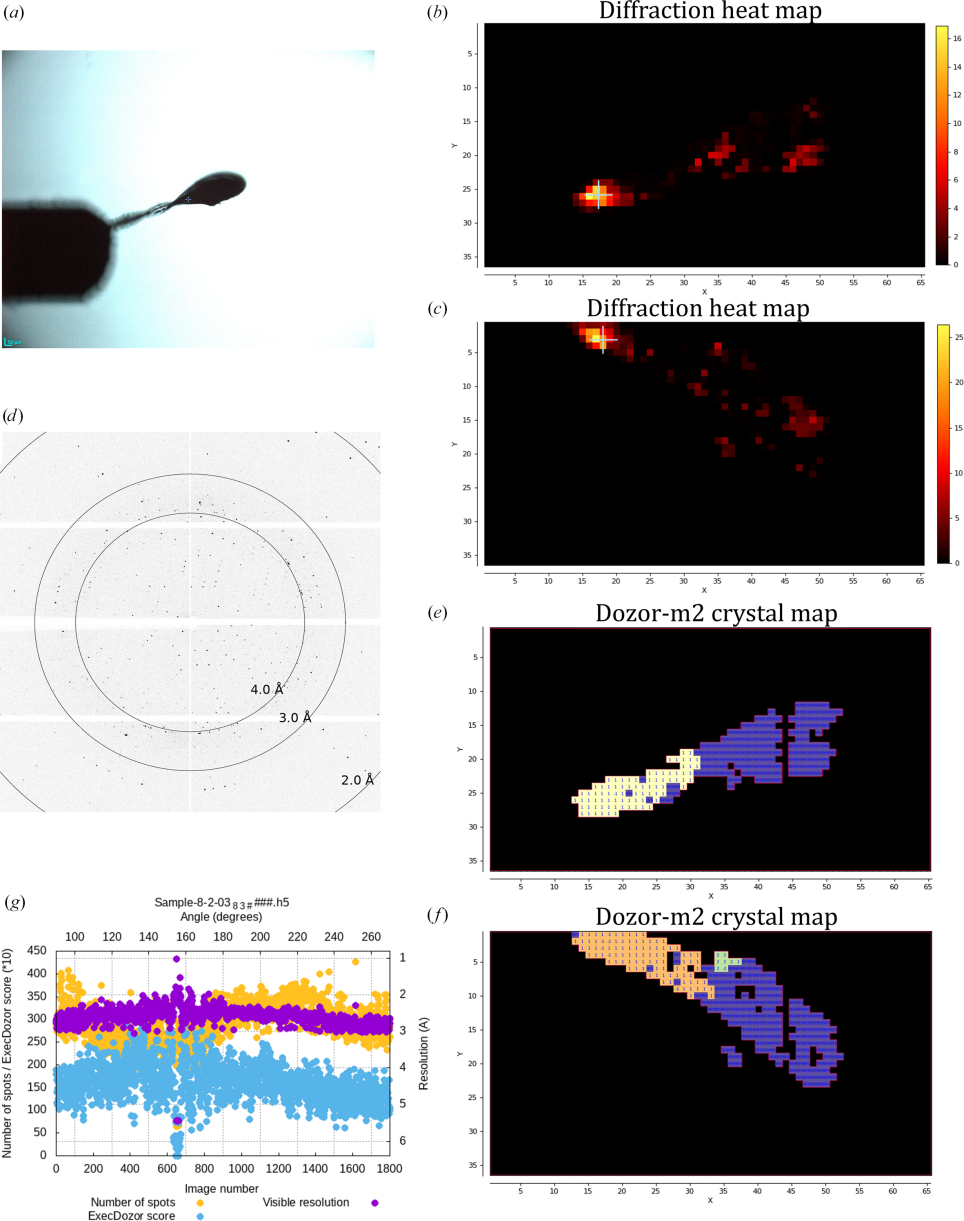
Data collection from a crystal of thermolysin diffracting to moderate resolution (∼2.5 Å). (*a*) A snapshot of a sample holder containing the crystal. (*b*, *e*) Two diffraction heat maps of the mesh scans separated by 90° of sample rotation. *X* and *Y* are the two axes of the scan; *X* is parallel to the goniometer rotation axis, whereas *Y* is the laboratory vertical axis. Units on the axes are scan steps, which are equal to the beam size (10 µm). (*c*, *f*) Two crystal maps for the mesh scans generated by *Dozor-m*2. Different colours are used to show distinct crystal areas, which are arbitrarily numbered, and the numbers are shown for each pixel; regions with overlapping diffraction patterns or contaminated by salt diffraction are shown in violet and numbered 999. (*d*) A sample diffraction image from the mesh scans. (*g*) Data-collection plot for the position selected by the method.

A crystal of thermolysin diffracting to ∼2.5 Å resolution was harvested in a standard sample holder (Fig. 6 ▸) and two mesh scans were performed separated by a 90° turn of the rotation axis. A beam aperture of 10 µm was used for the scans and the exposure time was 0.015 s per image (the dose per image was 35 kGy). *Dozor-m*2 found one and two individual crystal areas in the first and the second scan, respectively. From the only possible crystal pair combination on the two scans *Resheteau* confirmed this position to be truly matching crystals. Subsequently, 180° of rotation data were collected from this position. The diffraction images were processed with *Dozor*, *XDS* and *XSCALE* (Kabsch, 2010 ▸). The diffraction of the crystal was anisotropic with resolution limits ranging from 2.3 to 2.7 Å, hence the lack of completeness at a resolution of 2.3 Å. Data-collection statistics are given in Table 4 ▸.

## 4. Discussion

Despite the wide opportunities opened by serial crystallo­graphy methods (serial femtosecond crystallography and serial synchrotron crystallography), the conventional MX data-collection procedure remains indispensable for high-quality structure determination, as it usually offers a better data quality/quantity ratio. Accurate centring of crystals is a critical factor in ensuring the collection of optimal data. In this paper, we have presented a new combined approach for sample localization in MX based on the existing methods and software programs such as X-ray mesh scans with *Dozor* diffraction scoring. While the previously used workflows are suboptimal in crystal centring (X-ray centring uses a line scan to obtain the third coordinate of a crystal, which may misdirect to a different crystal in an orthogonal direction; the original *Mesh&Collect* has a limited oscillation range per crystal), our new approach allows 3D multi-crystal centring even in the case where the sample area contains multiple crystals. We believe that this can make a remarkable step forwards in sample characterization for MX and will be especially valuable for data-collection automation, ensuring the extraction of the maximum possible data quality and quantity from each sample. The method, however, inherits common limitations as a mesh-scan-based approach.

Depending on the quality of crystal diffraction, a higher radiation dose may be required to be absorbed by each crystal. *Dozor* is able to recognize the presence of diffraction from macromolecular crystals as small as a few micrometres. The exposure limit at which diffraction is detected depends on the X-ray beam parameters and both the crystal size and quality. As shown in Section 3.1, crystals with a size of around 25 µm or larger can be detected by exposing them to the beam such that the absorbed dose is less than 1 kGy; for crystals as small as 5 µm a dose around 50 kGy is necessary.

*Dozor-m*2 mesh-scan analysis is a useful tool for 2D crystal cartography. It has been successfully used for the last five years at the ESRF as a main analysis tool for the X-ray centring procedure. However, for 3D crystal cartography, a fundamental limitation arises on the crystal size. Because the diffraction is essentially anisotropic, proper 3D cartography of a large (larger than the size of the beam by at least an order of magnitude) crystal is challenging if not impossible. In the general case, the problem of 3D centring of a large crystal for standard data collection may not have an optimal solution at all. For example, the best regions of diffraction in orthogonal directions may be on completely opposite sides of a crystal. The resulting 3D centring point calculated may be outside the crystal and end up with the crystal going out of the beam, causing missing frames and incomplete data. Hence, a reasonable trade-off must be established between the desire to centre on a more homogeneous area of a large crystal and the risk of missing a portion of data. The proper deconvolution of the values of a diffraction-quality tensor will require multiple scans at different orientations, which are almost impossible to perform given the increasing radiation damage to the sample. For crystals with sizes comparable to the size of the beam, the above-mentioned problem is much less relevant.

The novel improvement that makes the *DoubleMesh&Collect* method work is the analysis carried out by the *Resheteau* program. It allows the recognition of crystals in different mesh scans by their diffraction patterns at known orientations, a problem that previously would have required an indexing algorithm, which is not particularly efficient on two still images from two mesh scans. The program is quite efficient even at a medium resolution of 2.0–3.0 Å; for poorly diffracting crystals at >3.0 Å with fewer data available for analysis the efficiency decreases. The angular difference between the scans is optimal at 90°; practical limitations, however, often necessitate smaller angles, with the accuracy decreasing as the angle narrows. With typically *N* = 300–1000 diffraction spots per image, the main computationally intensive parts of the algorithm are projection calculation, histogram sorting and several FFTs based on *N* spots for a set of 2500 unit vectors {*O*[2500(3*N* + *N*log*N* + *n*_bins_log*n*_bins_)] complexity}. Each crystal calculation can be parallelized, which makes the overall complexity of the algorithm ∼*O*(2500*N*log*N*), which can in principle be further decreased to *O*(*N*log*N*) by using a GPU-parallelization procedure. In fact, for the examples presented in this paper the total computational time on an ESRF server was between 5 and 20 s.

## 5. Code availability

The code implementation of *Resheteau* is available at https://github.com/neoswing/doublemesh/.

## Figures and Tables

**Figure 7:**
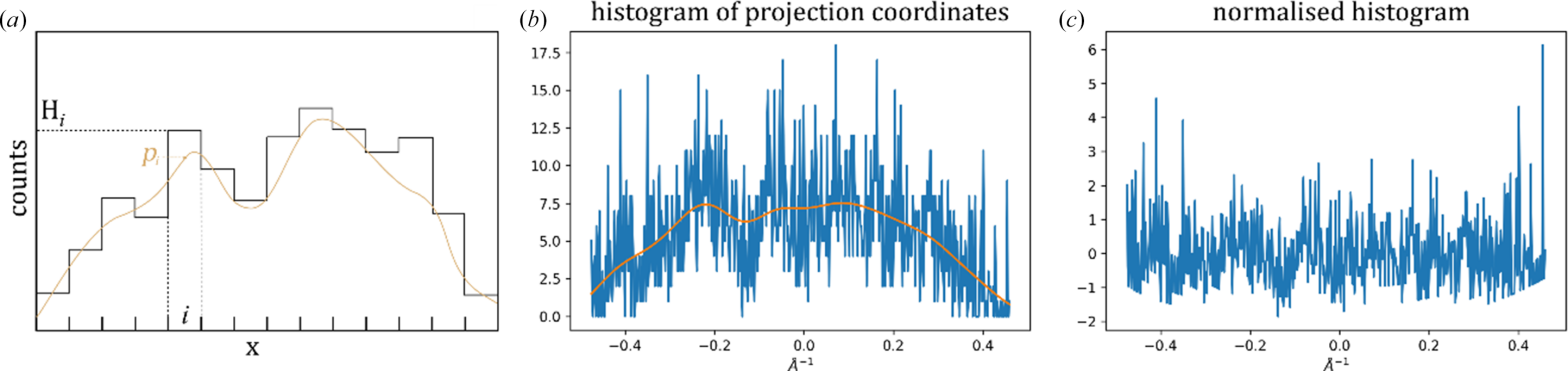
Histogram-normalization procedure. (*a*) Schematic illustration of a sample histogram with the number of counts *H*_*i*_ in the *i*th bin. We use a Gaussian filter (see text) to make a smooth averaging of the histogram (*p*_*i*_, shown in brown), which is then used for histogram normalization (see equation 6). (*b*) A sample histogram from mesh-scan diffraction data; the orange line indicates a smooth moving average of the histogram. (*c*) The normalized histogram from (*b*), using the moving average as the offset and its square root as a scale factor (see equation 6).

**Figure 8:**
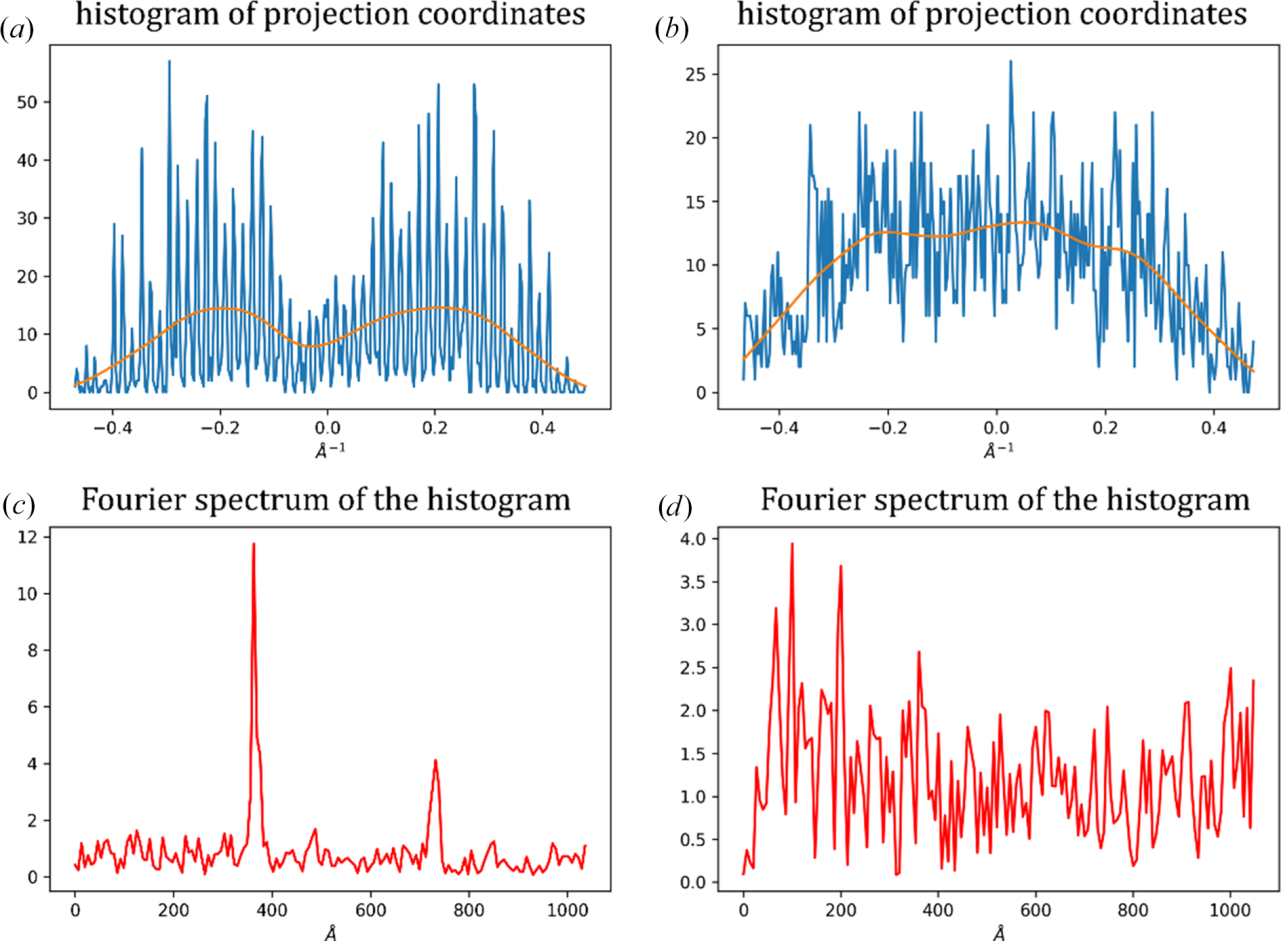
Determining periodicity in a histogram. (*a*) A sample histogram of projection coordinates with strong periodicity from a diffraction pattern in a mesh scan. The orange line represents a smooth average (see Fig. 7 ▸). (*b*) A sample histogram of projection coordinates without periodicity (within the Nyquist frequency) from a diffraction pattern in a mesh scan. The orange line represents a smooth average. (*c*) The Fourier spectrum of a normalized histogram from (*a*); the peak corresponding to the frequency of ∼370 Å is visible, as well as the doubling peak at ∼740 Å, which are outliers to the distribution. (*d*) The Fourier spectrum of a normalized histogram from (*b*); no distinct outliers are present in the distribution and the histogram is considered to be without any periodic pattern.

**Table 1:** Data-collection and processing statistics for MAR crystals measured using the *DoubleMesh&Collect* approach under cryogenic conditions

Crystal No.	Space group	*a*, *b*, *c* (Å), α, β, γ (°)	Resolution (Å)	Completeness (%)	Multiplicity	*R*_meas_ (%)	〈*I*/σ(*I*)〉
1	*R*3	89.6, 89.6, 148.2, 90, 90, 120	50–2.10 (2.23–2.10)	91.6 (91.7)	1.9 (1.8)	10.0 (208)	8.4 (1.1)
4			50–1.90 (2.02–1.90)	79.7 (80.3)	2.2 (2.1)	10.5 (174)	7.7 (1.0)
10			50–2.00 (2.13–2.00)	94.8 (96.6)	1.8 (1.8)	10.1 (217)	7.4 (0.7)
Merged (3 best)			50–1.90 (1.95–1.90)	100 (100)	5.2 (5.2)	12.0 (359)	10.0 (1.1)
Merged (16 in total)			50–1.90 (1.95–1.90)	100 (100)	21 (5)	16.3 (336)	17.1 (1.07)

**Table 2:** Data-collection and processing statistics for BR crystals measured using the *DoubleMesh&Collect* approach under room-temperature conditions

Crystal	Space group	*a*, *b*, *c* (Å), α, β, γ (°)	Overall completeness (%)	Multiplicity	*R*_meas_ (%), overall/low-resolution shell (60–9 Å)	〈*I*/σ(*I*)〉, overall/high-resolution shell (1.9–1.8 Å)	Resolution cutoff (〈*I*/σ(*I*)〉 = 1) (Å)
1	*C*222	118.5, 120.1, 37.0, 90, 90, 90	75	2.8	7.8/4.1	5.7/0.9	1.9
2			69	3.2	14.1/5.5	4.8/0.35	1.95
3			74	3.0	13.1/6.2	4.1/0.3	2.0
4			89	2.5	14.0/4.4	4.1/0.2	2.0
5			64	3.5	12.3/4.3	4.9/0.4	2.0
6			69	3.2	14.8/5.9	4.2/0.2	1.95
7			90	2.2	8.4/4.2	5.6/0.1	1.97
Merged			100	13.2	15.8/5.9	11.1/1.0	1.8

**Table 3:** Data-collection and processing statistics for horse myoglobin crystals measured using the *DoubleMesh&Collect* approach

Position of data acquisition	Space group	*a*, *b*, *c* (Å), α, β, γ (°)	Overall completeness (%)	Multiplicity	Overall *R*_meas_ (%)	〈*I*/σ(*I*)〉, overall/high-resolution shell (1.7–1.6 Å)	Resolution cutoff (〈*I*/σ(*I*)〉 = 1) (Å)
1-1	*P*2_1_	35.1, 28.7, 63.7, 90, 105.8, 90	64.6	1.6	6.5	4.6/0.6	1.7
1-2			60.3	1.8	4.3	9.9/1.5	1.6
2-1			67.2	1.6	6.8	5.1/0.7	1.7
2-2			64.1	1.7	8.1	4.3/0.3	1.85
All			96.5	4.4	15.5	5.6/1.1	1.6

**Table 4:** Data-collection statistics for the thermolysin crystal measured using the *DoubleMesh&Collect* approach

Space group	*a*, *b*, *c* (Å), α, β, γ (°)	Resolution (Å)	Completeness (%)	Multiplicity	*R*_meas_ (%)	〈*I*/σ(*I*)〉
*P*6_1_22	93.4, 93.4, 129.5, 90, 90, 120	50–2.27 (2.41–2.27)	91.7 (61.9)	14.8 (5.1)	11.0 (76)	19.4 (0.95)

## Appendix A

Let us consider a random sample diffraction pattern of a macromolecular crystal from a mesh scan (see, for example, Fig. 1 ▸*b*). We shall process the peak positions found by *Dozor* and construct a subset of the 3D lattice in reciprocal space (as shown in Section 2.3.1). Note that as the images recorded in a mesh scan are stills, we cannot unbiasedly estimate the centroid positions of reciprocal-lattice nodes as a systematic bias is always present; this, however, does not affect the convergence of the method. Despite the periodic arrangement of the node positions, if we inspect periodicity by projecting node coordinates to a random direction then most probably we will not see a periodic pattern in the resulting projection coordinates. Although there will always be one, the period will be too small to distinguish, given the limited subset size and the error and bias of centroid position estimates. We continue our analysis by constructing a histogram of projection coordinates of the nodes with the bin size providing a minimum of five counts per bin on average. The following probabilistic model is then assumed: we consider a Bernoulli scheme, in which each bin of the histogram has a certain probability *p_i_* of accommodating a node projection coordinate (see Fig. 7 ▸*a*). Without a periodicity in the histogram, the distribution *p_i_* of node coordinates across bins is considered to be continuous and not rapidly oscillating. We can therefore smoothen the histogram to obtain an approximation to the expected value *Np_i_* in each bin (see below), where *N* is the total number of diffraction spots. To do so, a moving window average with Gaussian weights (1D Gaussian filter) is used.

Given the large number of bins,

then the number of counts *H_i_* in each bin will follow the Poisson distribution,

where *N* is the total number of diffraction spots and σ^2^ is the variance. With *N* being large enough, by the central limit theorem

This means that the normalized histogram η_i_ will be asymptotically normally distributed. Let us derive the distribution on the Fourier amplitudes of the normalized histogram. For the discrete Fourier transform,

Each of the components, *A_k_* and *B_k_*, is a sum of independent standard normally distributed values η_j_, multiplied by trigonometric constant factors, then *A_k_* and *B_k_* are also normally distributed. Let us calculate variances for *A_k_* and *B_k_*:

Similarly,

By defining ,  and  now should follow the χ^2^-distribution and χ-distribution with two degrees of freedom, respectively, based on the facts that *c_k_A_k_* and *c_k_B_k_* are both standard normally distributed with *n*_bins_ being sufficiently large and that .

This justifies that if there is an outlier peak in the normalized Fourier spectrum *c_k_*|*F_k_*| (see Fig. 8 ▸), which might correspond to the real periodicity in projection coordinate arrangement, we can quantify the probability by estimating α, with the peak value being a (1 − α) quantile of the χ-distribution. The cumulative distribution function for an χ-distribution with two degrees of freedom (Rayleigh distribution) is

The (1 − α) quantile is

Thus, the estimate −logα = peak^2^/2 is now used in *Resheteau* as a quantitative indicator of periodicity.
